# Biliary stents reshape the bile microbiome in the absence of cholangitis

**DOI:** 10.1055/a-2733-3468

**Published:** 2025-11-11

**Authors:** Atsuto Kayashima, Seiichiro Fukuhara, Kentaro Miyamoto, Eisuke Iwasaki, Motohiko Kato, Tomohisa Sujino

**Affiliations:** 1 38547 Department of Gastroenterology and Hepatology, National Hospital Organisation Tokyo Medical Center, Meguro, Japan; 2 38084 Center for Diagnostic and Therapeutic Endoscopy, Keio University School of Medicine Graduate School of Medicine, Shinjuku, Japan; 3 599703 Research Department, R&D Division, Miyarisan Pharmaceutical Co. Ltd., Saitama, Japan; 4 38084 Division of Gastroenterology and Hepatology, Department of Internal Medicine, Keio University School of Medicine Graduate School of Medicine, Shinjuku-ku, Japan; 5 38084 Laboratory of Sakaguchi, Department of Multidimensional Analysis of Gastrointestinal Biology, Keio University School of Medicine Graduate School of Medicine, Shinjuku-ku, Japan

**Keywords:** Pancreatobiliary (ERCP/PTCD), Quality and logistical aspects, Hygiene, ERC topics

## Abstract

**Background and study aims:**

Biliary stents are widely used in endoscopic retrograde cholangiopancreatography (ERCP), yet their impact on the native bile microbiome under non-infectious conditions remains unclear. We aimed to characterize stent-associated alterations in the biliary microbiome using 16S rRNA gene sequencing.

**Patients and methods:**

We analyzed bile samples collected during ERCP from 35 patients without clinical or laboratory evidence of acute cholangitis. Patients were categorized into a control group (n = 25; naïve papillae) and an endoscopic biliary stenting (EBS) group (n = 10; previously stented). Microbial composition was assessed using high-throughput 16S rRNA sequencing after propensity score matching to balance background characteristics.

**Results:**

Beta diversity differed significantly between groups (PERMANOVA, *P* < 0.01), despite no significant differences in alpha diversity. The EBS group demonstrated increased relative abundance of *Firmicutes* and *Fusobacteriota* , and depletion of *Proteobacteria* . Notably, *Enterococcus* was significantly enriched in the EBS group (log fold change 6.74; *q* < 0.01), whereas *Sphingomonas* was reduced.

**Conclusions:**

Endoscopic biliary stenting is associated with distinct bile microbiome alterations, characterized by enrichment of *Enterococcus* species in clinically stable patients. These findings suggest that stents may predispose to opportunistic colonization, providing a potential mechanistic link to future cholangitis. Recognizing such preclinical dysbiosis may inform tailored antimicrobial strategies and future stent design.

## Introduction

Endoscopic biliary stenting is a cornerstone of therapeutic endoscopic retrograde cholangiopancreatography (ERCP), widely used in management of both benign and malignant biliary obstruction 1 . Although stents improve biliary drainage and alleviate symptoms, they are also known to be factors that have significant impact on the causative pathogens in cases of cholangitis 2 .

Human bile is not sterile. Recent advances in sequencing technologies, particularly 16S rRNA gene sequencing, have revealed a complex and diverse biliary microbiome in health and disease 3 . However, the effects of biliary stent placement on this microbial community in absent overt infection remain poorly understood. Previous studies have primarily focused on infected bile or stent occlusion 4 , limiting our understanding of how stents may drive subclinical microbial shifts. Such dysbiosis may serve as a precursor to stent-related infections, influence antibiotic susceptibility, or impact stent patency.

In this study, we aimed to characterize the impact of prior biliary stent placement on composition of the bile microbiome in patients undergoing ERCP without clinical evidence of cholangitis. We hypothesized that stent presence, even in clinically stable patients, leads to specific and detectable alterations in the bile microbiota.

## Patients and methods

### Study design and setting

This study was a post hoc analysis using a biliary microbiome database collected at Keio University Hospital between August 2020 and February 2023. It was approved by the Institutional Review Board (IRB No. 20190055) and adhered to the ethical principles outlined in the Declaration of Helsinki. All participants provided written informed consent prior to study enrollment.

### Study participants

The study included patients who required ERCP clinically and met the following inclusion criteria: 1 no clinical signs or symptoms of acute cholangitis defined as Tokyo guideline 5 ; 2) not fitting the diagnostic criteria for primary sclerosing cholangitis due to the previous report that showed characteristic microbiome was thought to be associated 6 ; 3) absence of recent antibiotic use within the last 30 days prior to ERCP; 4) no external or internal biliary drainage; and 5) no biliary duct operation.

Patients were divided into two groups: a control group with naïve papillae who underwent ERCP for the first time and an EBS group with prior endoscopic biliary stenting who were undergoing ERCP.

### Sample collection

During the ERCP procedure, bile samples were collected directly from the bile duct using a sterile catheter immediately after accessing the duct without use of prophylactic antibiotics. The collected bile was then transferred into sterile containers and immediately frozen at -80°C until further analysis.

### 16S rRNA sequencing and microbial profiling

Microbial profiling was performed using 16S rRNA gene sequencing targeting the V3-V4 hypervariable regions. DNA was extracted from bile samples using the reported protocol 7 . Amplification of the V3-V4 regions of the bacterial 16S rRNA gene was carried out using two-step tailed polymerase chain reaction method. Sequencing was conducted on an Illumina MiSeq platform (Reagent Kit v3), generating paired-end reads of 300 bp. After sequencing, raw reads were quality-checked and filtered using FASTX-Toolkit (ver. 0.0.14) and Sickle (ver. 1.33), trimming bases with quality less than 20 and outputting paired-end reads with lengths greater than 130 bp. Operational taxonomic units (OTUs) were assigned after removing chimeric and noise sequences with DADA2 plugin of Qiime2 (ver. 2024.10) and outputting representative sequences and amplicon sequence variant (ASV) tables, and taxonomic classification was performed with the SILVA (ver. 138) database at a 99%.

## Statistical analysis

Continuous variables were expressed as median and interquartile range (IQR) and compared using the Wilcoxon rank sum test. Categorical variables were expressed as proportions (%) and compared using Fisher's exact test. Propensity score was obtained by logistic regression model including following covariates: age, sex, disease, total bilirubin, status of gallbladder, taking ursodeoxycholic acid, and taking proton pomp inhibitors. Covariates were selected based on their potential clinical relevance and previously reported associations with bile microbiome composition. Propensity score matching (PSM) was performed with setting of 1:1 nearest-neighbor matching without replacement. To assess the balance of covariates, absolute standardized mean differences (ASMDs) were calculated and ASMDs < 0.1 were considered well balanced. Beta diversity was compared using permutational multivariate analysis of variance (PERMANOVA). Differences in microbial composition were assessed using the analysis of composition of microbiomes with bias correction (ANCOM-BC) method, with adjustments for multiple comparisons using the Holm method (showed as q value). *P* < 0.05 and q < 0.001 were considered statistically significant. All statistical analyses were performed using Qiime2 (ver. 2024.5) and R (ver. 4.41).

## Results

### Patient characteristics

A total of 35 patients were included in the study, with 25 in the control group and 10 in the EBS group. Ten paired cohort were obtained after PSM and almost all of variables were well balanced except for sex (ASMD = 0.2) ( Table 1 ).

**Table:** **Table 1** Baseline characteristics after propensity score matching.

**Characteristic**	**Control** **N = 10**	**EBS** **N = 10**	***P* value **	**SMD**
Age (years)	73 (70, 80)	65 (56, 88)	0.8	0.09
Female	4 (40%)	5 (50%)	> 0.9	0.2
Total bilirubin (mg/dL)	0.70 (0.60, 0.80)	0.75 (0.50, 0.90)	0.8	0.03
Direct bilirubin (mg/dL)	0.10 (0.10, 0.10)	0.10 (0.10, 0.10)	0.2	- ^*^
Presence of gallbladder	9 (90%)	9 (90%)	> 0.9	0
Ursodeoxycholic acid	2 (20%)	2 (20%)	> 0.9	0
Proton pump inhibitors	6 (60%)	6 (60%)	> 0.9	0
Disease			> 0.9	0.2
Gallstones related disease	5 (50%)	6 (60%)		
Non-biliary disease	5 (50%)	4 (40%)		
Data are presented as n (%) for categorical variables and median (interquartile range) for continuous variables. ^*^ Because it was not used as a covariate in propensity score matching, the SMD was not calculated. EBS, endoscopic biliary stenting; SMD, standardized mean difference.				

In the EBS group, all patients had plastic stents placed across the papilla. Median duration of stent placement before sampling was 77.5 days. Among these patients, four of 10 (40%) presented with cholangitis at time of initial ERCP when the biliary stent was placed. Three patients underwent stent exchange during this period, but none developed cholangitis.

### Alteration of diversity

**Fig. 1:**
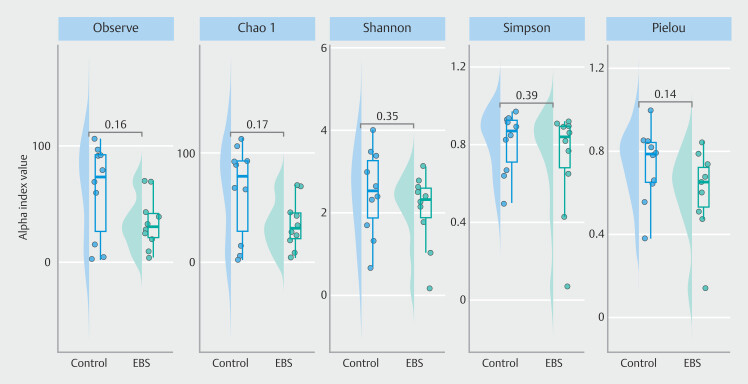
Alpha diversity of the bile microbiome in the control and EBS groups.

**Fig. 2:**
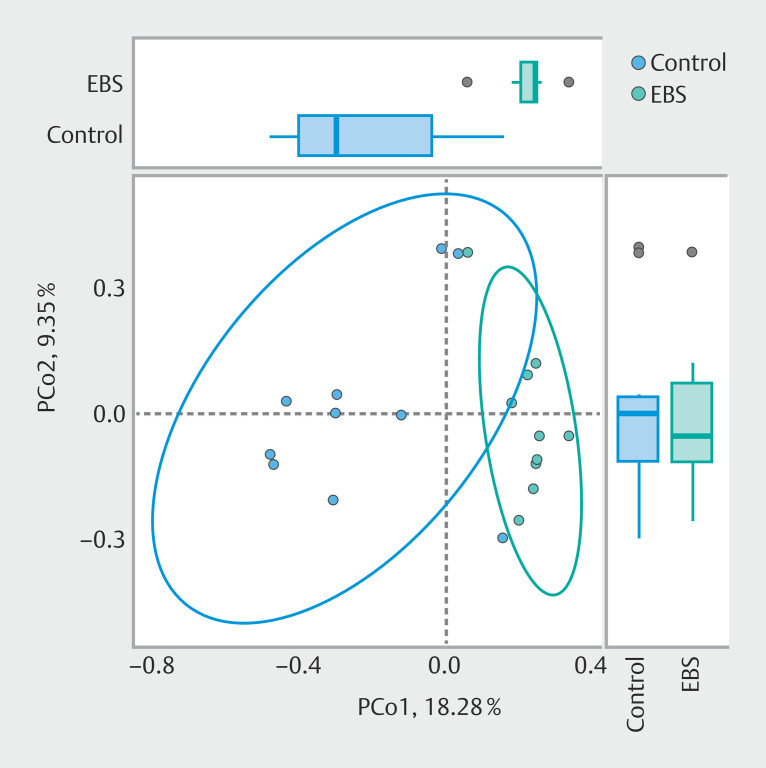
Beta diversity of the bile microbiome between the control and EBS groups.

Alpha diversity did not differ between the two groups, as measured by the observed features, Chao1 index, Shannon index, and Pielou’s evenness ( Fig. 1 ). Principal coordinates analysis (PCoA) plot of the Bray-Curtis dissimilarity revealed distinct clustering of microbial communities between the two groups ( Fig. 2 ). There was a significant difference in it, as assessed by PERMANOVA ( *P* < 0.01).

### Taxonomic composition

**Fig. 3:**
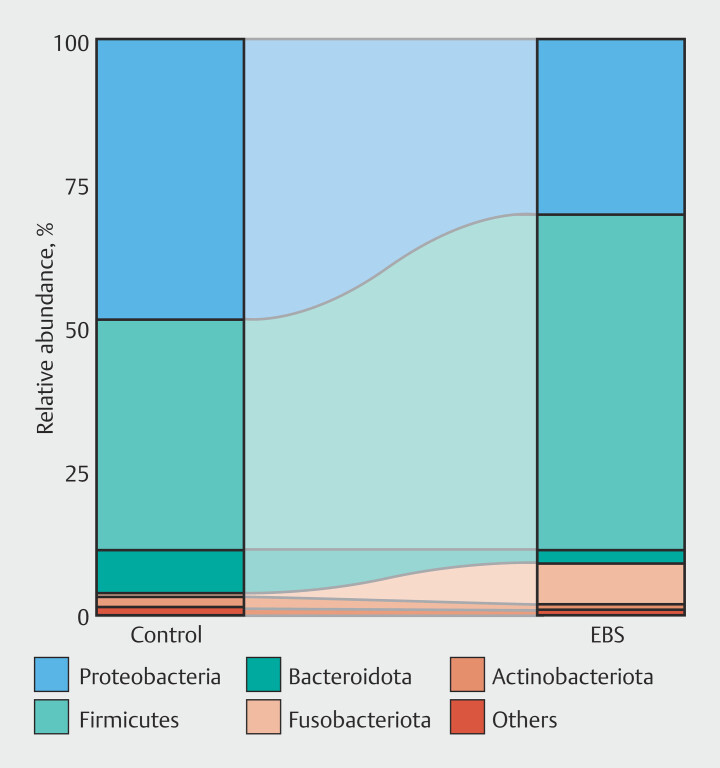
Relative abundance of dominant bacterial phyla in both groups.

**Fig. 4:**
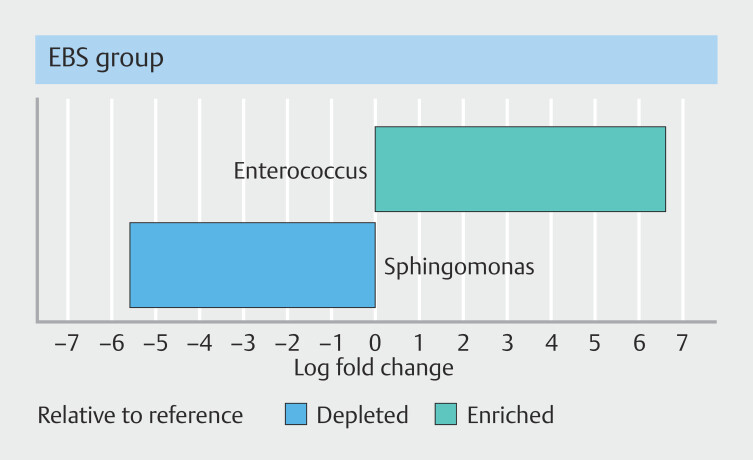
Differentially abundant genera identified by ANCOM-BC analysis.

At the phylum level, both groups were dominated by *Firmicutes* and *Proteobacteria* , but the relative abundance of specific taxa varied between the groups. In the EBS group, mean proportions of *Firmicutes* and *Fusobacteriota* were higher compared with the control group (40.0% vs. 58.4% and 0.5% vs. 7.4%, respectively) ( Fig. 3 ). Conversely, mean proportions of *Proteobacteria* , *Bacteroidota* , and *Actinobacteriota* were lower in the EBS group compared with the control group (48.4% vs. 29.9%, 7.6% vs. 2.1%, and 2.1% vs. 1.1%, respectively). ANCOM-BC analysis at the genus level revealed significant dysbiosis in the bile microbiome of the EBS group. Compared with the control group, the EBS group exhibited a significantly higher abundance of *Enterococcus* (6.74 log fold change; q < 0.01) and significantly lower abundance of *Sphingomonas* (-4.82 log fold change; q < 0.01) ( Fig. 4 ).

## Discussion

In this study, we demonstrated that prior biliary stent placement is associated with distinct changes in the bile microbiome, even in the absence of acute cholangitis. Specifically, patients with previously placed stents exhibited significant enrichment of *Enterococcus* and reduction in *Proteobacteria* , including *Sphingomonas* . These shifts occurred despite no clinical or laboratory evidence of infection at the time of bile sampling.

Our findings expand on prior studies that have focused on bile cultures during stent occlusion or cholangitis. Unlike those studies, our cohort was limited to patients without active infection, allowing us to isolate the effects of stents themselves on microbial composition. Enrichment of *Enterococcus* , which is a genus frequently implicated in stent occlusion and antibiotic resistance 8 , may represent a predisposing factor for future infection.

Mechanistically, biliary stents may alter bile flow, introduce foreign surfaces that promote biofilm formation 9 , or facilitate retrograde microbial migration from the duodenum 10 . These changes could select for specific taxa such as *Enterococcus* , which are capable of surviving in harsh, antimicrobial-rich environments and forming biofilms.

In contrast, reduction in *Sphingomonas* was observed. We note that *Sphingomonas* spp. are common environmental organisms in the biliary microbiome 11 and their depletion may reflect displacement by biofilm-forming taxa such as *Enterococcus* under altered bile flow caused by stents.

Limitations include the relatively small sample size, which may reduce power to detect subtler microbiome shifts, and the lack of longitudinal follow-up to determine the clinical consequences of these microbial alterations. To reduce bias, we employed PSM to reduce confounding due to clinical differences between groups. However, because there might be some unknown potentially relevant factors, the possibility of residual confounding due to unmeasured variables cannot be completely ruled out. There is also a possibility that stent-related factors such as stent type, placement method, and placement duration may influence the biliary tract microbial community; however, this was not examined in the present study. Furthermore, in addition to the small sample size, absence of functional metagenomic analysis limits our ability to determine whether the observed taxonomic shifts translate into functional alterations or contribute causally to infection risk.

## Conclusions

In conclusion, biliary stents significantly reshape the bile duct microbiome, promoting expansion of *Enterococcus* species even absent infection. Recognizing these preclinical microbial shifts may inform future strategies for infection prevention, stent design, and antimicrobial stewardship in patients undergoing biliary interventions.

## Notes

Correction **Biliary stents reshape the bile microbiome in the absence of cholangitis** Atsuto Kayashima, Seiichiro Fukuhara, Kentaro Miyamoto et al. Endoscopy International Open 2025; 13: a27333468. DOI: 10.1055/a-2733-3468 In the above mentioned article the name of an author has been corrected. Correct is: Seiichiro Fukuhara. This was corrected in the online version on 17.12.2025.
