# Correction: Biliary stents reshape the bile microbiome in the absence of cholangitis

**DOI:** 10.1055/a-2772-3924

**Published:** 2025-12-16

**Authors:** Atsuto Kayashima, Seiichiro Fukuhara, Kentaro Miyamoto, Eisuke Iwasaki, Motohiko Kato, Tomohisa Sujino

**Affiliations:** 1 38547 Department of Gastroenterology and Hepatology, National Hospital Organisation Tokyo Medical Center, Meguro, Japan; 2 38084 Center for Diagnostic and Therapeutic Endoscopy, Keio University School of Medicine Graduate School of Medicine, Shinjuku, Japan; 3 599703 Research Department, R&D Division, Miyarisan Pharmaceutical Co. Ltd., Saitama, Japan; 4 38084 Division of Gastroenterology and Hepatology, Department of Internal Medicine, Keio University School of Medicine Graduate School of Medicine, Shinjuku-ku, Japan; 5 38084 Laboratory of Sakaguchi, Department of Multidimensional Analysis of Gastrointestinal Biology, Keio University School of Medicine Graduate School of Medicine, Shinjuku-ku, Japan



In the above mentioned article the name of an author has been corrected. Correct is: Seiichiro Fukuhara. This was corrected in the online version on 17.12.2025

